# Corrigendum: A comparison of qPCR and microscopy for the detection and enumeration of Cryptosporidium oocysts from drinking water

**DOI:** 10.1099/jmm.0.001926

**Published:** 2024-10-23

**Authors:** Guy Robinson, Kristin Elwin, Matthew Jones, Rachel M. Chalmers

**Affiliations:** 1 Cryptosporidium Reference Unit, Public Health Wales, Swansea, UK; 2 Swansea University Medical School, Swansea, UK; 3 Dŵr Cymru Welsh Water, Glaslyn, The Avenue, Coedkernew, Duffryn, Newport, UK

In the published version of this article, the reverse JR primer sequence in the Methods section was published incorrectly as JR 5′ CCTTACTCCTTCAGCACCTTA.

The correct reverse JR primer sequence is JR 5′-TAAGGTGCTGAAGGAGTAAGG.

The fully corrected statement reads as follows:

“The PCR assay [17] comprised the following primers (Integrated DNA Technologies): forward primers in combination, both at 600 nM final concentration, JF1 5′-AAGCTCGTAGTTGGATTTCTG and JF2 5′-AAGCTCGTAGTTAATCTTCTG (correspondent with position 601–621 GenBank L16996 C. parvum); reverse primer at 600 nM final concentration JR 5′-TAAGGTGCTGAAGGAGTAAGG (correspondent with position 1015–1035 GenBank L16996 C. parvum).

The authors apologise for any inconvenience caused.

